# Mild Pancreatitis Induced by Linagliptin Revealed by a Medication Review

**DOI:** 10.7759/cureus.36455

**Published:** 2023-03-21

**Authors:** Harish Gidda, Inderpal Singh, Ayman Mohamed, Bola Nashed

**Affiliations:** 1 Internal Medicine, Ascension St. John Hospital, Detroit, USA

**Keywords:** dipeptidyl peptidase-4, type 2 diabetes mellitus, pancreatitis, medication side-effects, linagliptin, dpp-4 inhibitors, pancreatitis causes, linagliptin pancreatitis

## Abstract

As dipeptidyl peptidase-4 inhibitors are becoming more utilized in the treatment of diabetes, it is important to recognize their side effects and become more familiar with them. As these side effects arise, physicians are more prepared to recognize and discontinue these medications. This case report describes a 34-year-old male who initially presented with a hemoglobin A1c greater than 16%. After titration of his diabetic medications, he presented with pancreatitis diagnosed by symptoms and imaging. Common causes of pancreatitis were ruled out, including biliary pathology, alcohol use, tobacco use, elevated calcium levels, and hypertriglyceridemia. The patient followed up in the clinic with persistent symptoms. A review of his medication list revealed pancreatitis as a side effect of linagliptin. After holding this medication, his symptoms improved over the course of a month.

## Introduction

Dipeptidyl peptidase-4 (DPP-4) inhibitors act by preventing the inactivation of incretin hormones such as glucagon-like peptide-1 (GLP-1). This increases glucose-dependent insulin release by the pancreas [1]. Linagliptin, one of the DPP-4 inhibitors, has been reported as a rare cause of acute and chronic pancreatitis [2]. The mechanism is unknown. There have been case reports describing pancreatitis caused by DPP-4 inhibitors [2-4]. Pancreatitis caused by DPP-4 inhibitors may be as rare as one in 1,000 cases, as reported by the government of the United Kingdom [5]. This case report follows a 34-year-old male who developed linagliptin-induced pancreatitis. It is important to become familiar with the side effects of DPP-4 inhibitors as they are becoming more utilized in the treatment of diabetes. Further awareness of pancreatitis as a side effect of linagliptin can expedite the discontinuation of this medication, leading to an accelerated recovery of linagliptin-induced pancreatitis.

## Case presentation

The following is a case report of a 34-year-old male with a past medical history of type 2 diabetes mellitus, hypertension, ischemic cardiomyopathy, and obesity, who originally presented to our outpatient clinic with a hemoglobin A1c greater than 16% (<5.7%). Linagliptin was added to Levemir and empagliflozin for further glycemic control. This combination improved his hemoglobin A1c to 9.2%. Approximately five months later, the patient presented to the emergency department (ED) with a two-week history of epigastric dull abdominal pain. He had one episode of non-bloody, non-bilious emesis, and five episodes of loose stools prior to arriving at the emergency room. He also reported ongoing nausea and worsening abdominal pain with oral intake over this time frame. Lab work revealed a mildly elevated lipase of 71 IU/L (13-60 IU/L). Total bilirubin (0.4 mg/dL; 0-1.5 mg/dL), alkaline phosphatase (63 IU/L; 20-120 IU/L), aspartate transaminase (28 IU/L; 0-45 IU/L), alanine transaminase (38 IU/L; 0-45 IU/L), and calcium (9.2 mg/dL; 8.4-10.5 mg/dL) were within normal limits. His triglyceride level was 209 mg/dL (30-149 mg/dL). Results can be seen below in Table 1. Fecal calprotectin level was drawn at this time and found to be 110 ug/g.

**Table 1:** Lab values and reference ranges

Lab	Value	Reference range
Hemoglobin A1c - initial	>16%	<5.9%
Hemoglobin A1c - repeat	9.2%	<5.9%
Lipase	71 IU/L	13-60 IU/L
Total bilirubin	0.4 mg/dL	0-1.5 mg/dL
Alkaline phosphatase	63 IU/L	20-120 IU/L
Aspartate transaminase	28 IU/L	0-45 IU/L
Alanine transaminase	38 IU/L	0-45 IU/L
Calcium	9.2 mg/dL	8.4-10.5 mg/dL
Triglyceride	209 mg/dL	30-149 mg/dL
Fecal calprotectin	110 ug/g	0-120 ug/g
White blood cell count	7.53 K/uL	4-11 K/uL
Red blood cell count	5.86 M/uL	4.5-5.9 M/uL
Hemoglobin	15.8 g/dL	13.5-17.5g/dL
Platelet count	165 K/uL	150-400 K/uL
Absolute polymorphonuclear cells	2.89 K/uL	1.8-7.5 K/uL
Absolute lymphocytes	3.77 K/uL	1-5 K/uL
Absolute monocytes	0.51 K/uL	0-1 K/uL
Absolute eosinophils	0.24 K/uL	0-0.4 K/uL
Absolute basophils	0.09 K/uL	0-0.2 K/uL

**Figure 1:**
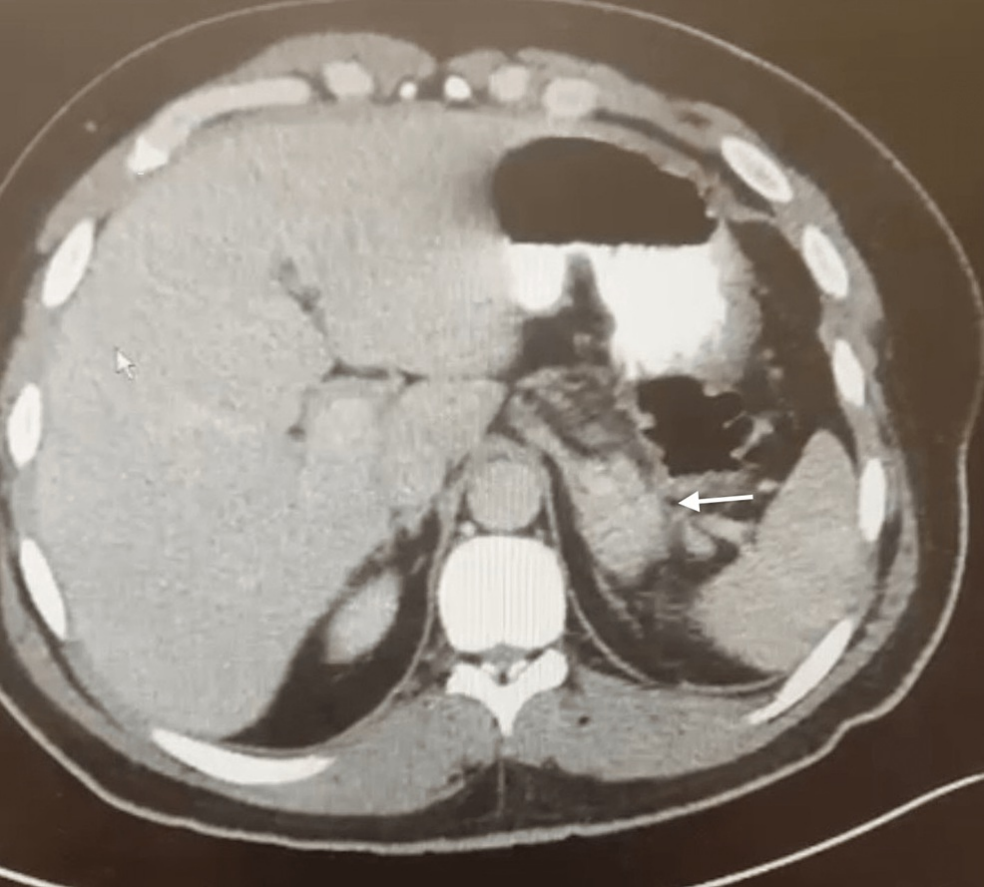
CT of the abdomen with IV contrast showing faint peripancreatic fat stranding adjacent to the pancreatic tail

He was a lifelong non-smoker and did not drink alcohol. A computed tomography (CT) scan with IV contrast of his abdomen showed faint peripancreatic fat stranding adjacent to the pancreatic tail, consistent with mild pancreatitis (Figure 1).

There was no evidence of cholelithiasis, gallbladder inflammation, or common bile duct dilatation. He was discharged from the ED and continued on his home medications, including linagliptin. At the post-hospital follow-up visit, the patient reported minimal improvement since his ED discharge and persistent nausea and epigastric discomfort with eating. The fecal calprotectin level was at 110 ug/g, which would typically warrant a repeat, but the patient had filled two out of three criteria for pancreatitis making it the most probable etiology of his symptoms. A search for the cause of pancreatitis began by reviewing the medication list. He was taking aspirin, atorvastatin, carvedilol, furosemide, lisinopril, Levemir, empagliflozin, and linagliptin. After a careful review of his medications and potential side effects, atorvastatin, lisinopril, and linagliptin were noted as potential causes of acute pancreatitis. The patient was taking atorvastatin and lisinopril for eight years prior to this acute pancreatitis event with no change in dosing for seven years. Linagliptin was the most recent addition to his medication regimen; thus, the clinical decision was made to discontinue this medication as the most likely medication causing pancreatitis. At a one-month follow-up visit, the patient reported improvement within one week since the discontinuation of linagliptin, with complete resolution of his nausea and abdominal pain in approximately two weeks. It was concluded that the patient’s pancreatitis was most likely due to the use of linagliptin, as he improved with discontinuation, and other typical causes were ruled out.

## Discussion

The patient presented to the emergency department with abdominal pain and nausea that were both worsened with oral intake over two weeks, with one episode of emesis and five loose bowel movements. The initial top differential diagnoses included gastroenteritis, peptic ulcer disease, and pancreatitis. Although the patient's lipase level was only mildly elevated at 71 IU/L, he had the signs and symptoms of pancreatitis, which were epigastric abdominal pain worsened with oral intake. These symptoms with the elevated lipase level and prolonged duration of symptoms placed pancreatitis higher on the differential leading to the CT of the abdomen/pelvis. The CT revealed findings consistent with pancreatitis with peripancreatic fat stranding of the tail. The patient had the signs and symptoms of pancreatitis and imaging findings consistent with pancreatitis, fulfilling two out of three criteria for pancreatitis, thus making the diagnosis of pancreatitis [6]. As it was thought the patient only had a mild case of pancreatitis, he was discharged home to follow up in the clinic.

On initial follow-up after the acute emergency department visit, the patient had persistent symptoms, which prompted the search for other etiologies of pancreatitis. After reviewing all the medications the patient was taking, atorvastatin, lisinopril, and linagliptin were noted to be the three potential medications that could cause pancreatitis [2-4,7-8]. Although atorvastatin-induced pancreatitis can happen at any time, it was more common a few months after initiation [8]. The patient had been taking lisinopril and atorvastatin for eight years prior to this episode, with no change in dosing for the past seven years. Linagliptin was the most recent addition to his medication regimen, and the clinical decision was made to stop this medication and monitor for the resolution of symptoms. The patient's symptoms started to improve after one week of holding the medication, with a resolution after two weeks.

This case report highlights a rare complication of linagliptin, which is drug-induced pancreatitis. It also highlights the importance of a careful medication review for possible side effects. The general class of DPP-4 inhibitors is associated with an increased risk of pancreatitis [9]. The CARMELINA study also found that there was a higher incidence of pancreatic cancer in patients who were taking linagliptin compared to those who were on placebo [10]. A possible mechanism for these complications may be the increase in GLP-1. The increase in GLP-1 has been observed to cause pancreatic duct gland expansion in animal autopsies [11]. When suspecting drug-induced pancreatitis, it is important that the most common causes are ruled out [12]. Such as in our case, biliary pathology, hypertriglyceridemia, hypercalcemia, and alcohol use were ruled out as possible etiologies.

Drug-induced pancreatitis may be a delayed diagnosis as clinicians may be unaware of pancreatitis as a rare potential side effect of many common medications. Angiotensin-converting enzyme inhibitors, 3-hydroxy-3-methylglutaryl coenzyme A (HMG-CoA) reductase inhibitors (statins), oral contraceptives, diuretics, antiretrovirals, and hypoglycemic agents are some of the common classes of medications used every day by clinicians, which may cause pancreatitis [13]. The side effect of pancreatitis is better known in some medications than others but still remains rare. It is important for physicians to recognize pancreatitis as a side effect of DPP-4 inhibitors and other common medications. Although rare, drug-induced pancreatitis may occur. The more appreciation and awareness clinicians have for drug-induced pancreatitis, the quicker the medication can be discontinued and expedite resolution.

## Conclusions

As medicine is advancing, newer drugs are emerging with better efficacy in treating common conditions such as type 2 diabetes mellitus. One of these medications is DPP-4 inhibitors, such as linagliptin. This was a case report of drug-induced pancreatitis caused by linagliptin. When a patient presents with pancreatitis, it is important to rule out the most common etiologies. After these have been ruled out, it is appropriate to start looking for other causes of pancreatitis, including medications, although typically rare. Many common medications have the potential to cause drug-induced pancreatitis. It is important that physicians are aware of the medications their patients are taking and their potential side effects. Reporting this case of linagliptin-induced pancreatitis serves as an important part of the medical literature to create more awareness of DPP-4-induced pancreatitis. Thus, allowing more rapid discontinuation of the DPP-4 inhibitor and expediting pancreatitis resolution.
